# Targeting *UCHL1* Induces Cell Cycle Arrest in High-Risk Multiple Myeloma with t(4;14)

**DOI:** 10.3389/pore.2021.606567

**Published:** 2021-03-31

**Authors:** Parin Kamseng, Teerapong Siriboonpiputtana, Teeraya Puavilai, Suporn Chuncharunee, Karan Paisooksantivatana, Takol Chareonsirisuthigul, Mutita Junking, Wannasiri Chiraphapphaiboon, Pa-thai Yenchitsomanus, Budsaba Rerkamnuaychoke

**Affiliations:** ^1^ Department of Pathology, Faculty of Medicine Ramathibodi Hospital, Mahidol University, Bangkok, Thailand; ^2^ Department of Medicine, Faculty of Medicine Ramathibodi Hospital, Mahidol University, Bangkok, Thailand; ^3^ Siriraj Center of Research Excellence for Cancer Immunotherapy (SiCORE-CIT), Faculty of Medicine Siriraj Hospital, Mahidol University, Bangkok, Thailand

**Keywords:** *UCHL1*, t(4;14) multiple myeloma, cell cycle regulation, biomarker, expression profile

## Abstract

Multiple myeloma (MM) patients considered to be at high cytogenetic risk commonly fail to respond to standard treatment. A thorough understanding of the molecular mechanism of MM development is, therefore, needed. We endeavored to explore the transcriptional signature among different subgroups of newly diagnosed MM using gene chip-based expression microarray. Bone marrow samples of 15 newly diagnosed Thai MM patients were included. The chromosomal translocation t(4;14) was the most frequently identified genetic alteration in the high-risk subgroup. Cluster analysis from expression profiling demonstrated that high-risk MM have a distinctly different expression pattern compared to standard-risk patients. The most significant differentially expressed gene was *UCHL1*. Functional enrichment analysis by Gene Set Enrichment Analysis, FUNRICH, and Gene Ontology Panther pathway revealed the gene sets involved in cell cycle control to be enriched in the t(4;14) high-risk group. Interestingly, among the well-established downstream targets of *UCHL1*, only *CCND2* was significantly expressed in the t(4;14) high-risk group. Suppression of *UCHL1* protein level by LDN-5744 inhibitor could arrest the cell cycle in G1 phase in cell lines. These findings shed light on the molecular mechanism of *UCHL1* in t(4;14) high-risk MM and support the evidence that alteration of the *UCHL1* pathway may play a role in the pathogenesis of high-risk MM.

## Introduction

Multiple myeloma (MM) or plasma cell myeloma (PCM) is a terminally differentiated B-cell neoplastic disorder that is characterized by the presence of clonal proliferation of malignant plasma cells (PCs) in bone marrow, and excessive monoclonal immunoglobulin, which is associated with multiple organ dysfunction [1]. MM was the second most common cancer of the hematopoietic and reticuloendothelial systems according to registration data from the Ramathibodi Cancer Report 2017, which was published by Ramathibodi Hospital, Bangkok, Thailand [2]. Even though novel therapeutic approaches have profoundly improved the overall survival of MM patients, drug resistance and treatment failure still occur [3]. Improved understanding of the underlying molecular mechanism of MM pathogenesis is, therefore, needed so that more effective treatments can be developed.

Several previous studies have attempted to investigate the molecular mechanisms underlying the pathogenesis of MM, including disease progression, tumor expansion, metastasis, treatment response, and MM drug resistance [4–7]. Microarray, which is one of many advanced technologies in cellular and molecular genetics, has yielded information and understanding about the clonal evolution, pathogenesis, and progression of MM, and this has led to the identification of biomarkers that can be used to develop potential targeted drug therapies [5, 8, 9]. Moreover, the identification of prognostic factors is urgently needed in the high-risk subgroup of MM to improve treatment protocols and outcomes.

Primary cytogenetic abnormalities are currently the major markers used to classify high-risk disease [i.e., t(4;14), del(17/17p), t(14;16), t(14;20), non-hyperdiploidy, and gain(1q)], and there are many tools that can be employed to identify the subgroup of MM, including expression profiling. The primary cytogenetic alteration t(4;14) was found to be a high cytogenetic risk factor for MM with the prevalence of 6–12% [10]. Previous studies reported MM with chromosomal translocation t(4:14) to be associated with high expression of *UCHL1* [11]. The deubiquitinating enzyme UCHL1, which is a member of the ubiquitin carboxy terminal hydrolase family, catalyses the hydrolysis of C-terminal ubiquitin esters and amides to regulate the protein degradation system [12]. Alterations in ubiquitin-proteasome pathways have been reported in several diseases, such as neurodegenerative disorders [13, 14] and particular types of cancers [15, 16]. In hematologic malignancy, *UCHL1* was found to be overexpressed in mature B cell malignancy, including aggressive germinal center diffuse large B-cell lymphoma [17], and to be an aggressive biomarker in MM development [11]. The role of *UCHL1* in cancer is complicated by the fact that it can act as an oncogene via many molecular mechanisms depending on the cancer type. For example, *UCHL1* can stabilize and upregulate β-catenin/TCP-dependent transcription in colorectal cancer [18], can promote metastasis as a deubiquitinating enzyme for *HIF-1a* in lung cancer [19], can bypass the need for mTORC1-dependent protein synthesis to initiate *MYC* translation in lymphomas [20], and can enhance CDK activities in the pathogenesis of neurodegenerative diseases [21]. Additionally, *UCHL1* was found to be a poor biomarker in aggressive MM due to its requirement for disease progression [11]; however, our understanding of the molecular mechanism of *UCHL1* in MM is still unclear.

In this study, we aimed to use microarray-based technology to explore the gene expression signature in MM patients with high cytogenetic risk compared to that of MM patients with standard cytogenetic risk according to the cytogenetic risk classification system published by the International Myeloma Working Group 2016 [22]. We found that MM patients with t(4;14) had differential overexpression of *UCHL1* compared to non-t(4;14) MM group. Functional enrichment analysis indicated that cell cycle gene sets were enriched in the t(4;14) high-risk group of MM. Additionally, protein-protein interaction demonstrated that *UCHL1* can regulate the cell cycle via downstream target *CCND2*, and overexpression of *CCND2* was observed in the t(4;14) high-risk group of MM when compared with non-t(4;14) MM group. Targeting *UCHL1* in KMS11 and KMM1 myeloma cell lines could suppress the expression of the *UCHL1* protein resulting in G1 phase arrest in MM cell lines. The findings of this study support and suggest the potential use of *UCHL1* as a biomarker, and as a promising therapeutic target in patients with high-risk MM.

## Materials and Methods

### Samples

Three to five millilitres of bone marrow samples was collected from leftover specimens from the Human Genetic Laboratory, Department of Pathology, Faculty of Medicine Ramathibodi Hospital, Mahidol University, Bangkok, Thailand during February 2017 to October 2018. Bone marrow samples of 15 newly diagnosed MM patients were divided into two groups. Five samples were allocated to the high cytogenetic risk MM group, and the other 10 samples were assigned to the standard cytogenetic risk MM group. Among those samples, four of five samples in high-risk group were subdivided into t(4;14) and non-t(4;14) subgroups in order to perform the functional enrichment analysis comparison. Group classification was determined according to the criteria set forth in the International Myeloma Working Group consensus multiple myeloma with high-risk cytogenetics 2016. The clinical and laboratory features including serum β_2_-microglobulin (β_2_M), serum creatinine, serum hemoglobin, follow-up time and clinical response were collected from medical records. The clinical and laboratory features of 15 MM patients were shown in Table 1.

**TABLE 1:** Biological characteristics of multiple myeloma (MM) samples.

No.	Genetic alterations	Age (years)	Serum β_2_M (mg/L)	Serum creatinine (mg/dl)	Serum Hb (g/dl)	R-ISS staging	Follow-up time (months)	Clinical response
Standard cytogenetic risk samples								
1	4p16(FGFR3)×3	64	3.33	0.86	8.5	II	42	CR
2	del(13), CCND1×4	58	3.94	0.76	12.7	II	36	VGPR
3	(TP53, D17Z1) ×1	55	1.73	0.79	12.7	I	33	CR
4	del(13q14.3)	80	5.44	2.4	9.5	II	Loss FU	Loss FU
5	CCND1×3	64	5.55	0.63	9	III	33	VGPR
6	IGH×1, CCND1×3	77	6.21	6.01	8.4	III	29	CR
7	CCND1×3	51	1.16	1.27	10.4	II	32	PR
8	del(13q14.3)	71	3.29	1.12	10.7	I	31	Relapsed
9	Trisomy(13), 1p36×2, (FGFR3, CCND1, CCND3, MAFB, IGH) ×3	65	2.00	0.68	9.8	I	30	VGPR
10	TP53*3	77	3.27	0.69	9.6	II	30	CR
Median serum β_2_M			3.59 mg/L
Median serum creatinine			1.52 mg/dl
Median serum hemoglobin			10.13 g/dl
High cytogenetic risk samples								
11	del(13), del(17p13.1), t(4;14), FGFR3*3, IGH*3, TP53*1	87	8.72	1.47	8.2	III	Loss FU	Refuse treatment
12	del(13), del(17p13.1), del(FGFR3), SRD*4, CCND3*3, del(MAF)	59	7.05	1.19	7	III	2	Death
13	del(13), t(4;14)	69	11.1	2.59	9	III	Loss FU	Loss FU
14	Trisomy(17), t(4;14)	52	4.29	0.94	7	III	23	PR
15	del(13q14.3), t(4;14)	77	9.53	1.07	9.2	III	5	VGPR
Median serum β_2_M			8.14 mg/L					
Median serum creatinine			1.45 mg/dl					
Median serum hemoglobin			8.08 g/dl					
Median age			65 years					
Median follow-up time			30 months					

*Reference values: β_2_-microglobulin: 0.7–3.4 mg/L, Creatinine: 0.55–1.02 mg/dl, Hemoglobin: 12.00–16.00 g/dl.

β_2_M, β_2_-microglobulin; Hb, hemoglobin; R-ISS, revised multiple myeloma international staging system; CR, complete response; VGPR, very good partial response; Loss FU, lost to follow-up; PR, partial response.

### Cell Collection and Total RNA Extraction

Plasma cells (PCs) were isolated from bone marrow samples by immunomagnetic bead selection with monoclonal mouse antihuman CD138 antibodies using a magnetic column (Miltenyi Biotec, Bergisch Gladbach, Germany) according to the manufacturer’s instructions. PC purity was confirmed by MACs technique as greater than 95% CD138 + cells and morphology by Wright-Giemsa staining. RNA was extracted using a mirVana™ miRNA Isolation Kit (Life Technologies, Carlsbad, CA, United States), and its concentration was determined using a NanoDrop 2000 (Thermo Fisher Scientific, Waltham, MA, United States) according to the manufacturer’s instructions.

### Gene Expression Profiling and Functional Enrichment Analysis

Gene expression profiling was performed on an Affymetrix GeneChip Human Clariom S Pico Assay (Affymetrix, Santa Clara, CA, United States). All CEL files were analyzed using Transcriptome Analysis Console (TAC) 4.0 software (Thermo Fisher Scientific). Expression analysis to determine genes that significantly differentially express was performed using analysis of variance (ANOVA). Significant differential expression was defined as gene level fold change less than −2 or more than 2, and a *p*-value < 0.05. A Probeset (Gene/Exon) was considered expressed if ≥ 50% of samples had a detection above background (DABG) value below the DABG threshold (DABG <0.05). Functional enrichment analysis was performed using 3 types of software. including Gene Set Enrichment Analysis (GSEA), FUNRICH, and Gene Ontology (GO) Panther pathway. For GSEA, a collection of annotated gene sets was assembled and uploaded into Molecular Signatures Database version 7.0 (updated August 2019), which was used as a gene sets database. The chip platform used in this study was Clariom S Human HT.r1.chip. The weighted enrichment statistic was used to analyze the expression profile of each sample. The metric for ranking genes was set as ratio of classes. Genes from expression profile analysis were evaluated using FunRich software, and there were 941 of 960 genes mapped in the FUNRICH database. Hypergenometric *p*-value test was performed against all 20,515 genes in the FunRich database. All of the 960 genes identified from gene expression profiling were analyzed in the GO Panther pathway. Over-representation test was performed using version 14.1 PANTHER annotation platform. All genes in the database of Homo sapiens were selected as the reference list. Fisher's exact test was used to compare data from the PANTHER pathways annotation dataset. Protein-protein interaction (PPI) network was created using STRING database version 1 [23]. We used search tool for multiple proteins by name to analyze the PPI network.

### Cell Culture and DNA Index

MM cell lines, including KMM1 and KMS11, were kindly provided by Professor Seiji Okada of the Center of AIDS Research, Kumamoto University, Kumamoto, Japan. All cell lines were cultured in Roswell Park Memorial Institute (RPMI)-1640 medium supplemented with 15% fetal bovine serum (FBS) (Thermo Fisher Scientific) and 100 U/ml penicillin-streptomycin at 37°C in a humidified atmosphere containing 5% CO_2_. All cell lines were treated with UCHL1 inhibitor LDN-5744 (Sigma-Aldrich Corporation, St. Louis, MO, United States) at concentrations of 10, 20, and 40 ng/μl for 24 h and DMSO (AMRESCO) as control. These doses were selected based on previous IC50 studies that showed a specific inhibition of the proteasome activity for UCH-L1 [24]. Cells were then counted using trypan blue staining. The cells obtained from both treated and untreated conditions at about 1 × 10^6^ cells were analyzed for DNA index by propidium iodide (PI) staining. The cells were centrifuged at 450 g for 3 min and then the supernatant was discarded. After that, 1 µl of PI/RNase staining solution (BD Biosciences, Franklin Lakes, NJ, United States) and 1 µl of Triton X-100 solution (Fluka Analytical, Buchs, Switzerland) were added and incubated for 20 min. The cells were then analyzed using a Cytomics FC 500 flow cytometer (Beckman Coulter, Brea, CA, United States). Independent-samples *t* test was used as statistical testing of cell counting by trypan blue staining and DNA index.

### Real-Time Polymerase Chain Reaction

Total RNA was reverse transcribed into cDNA using a SuperScript VILOTM cDNA Synthesis Kit (Thermo Fisher Scientific). Real-time quantitative reverse transcription polymerase chain reaction (RT-qPCR) was performed using Express SYBR GreenER™ qPCR SuperMix (Life Technologies) and normalized to RNA level by *GAPDH*. The primer sequences were, as follows: *UCHL1*—sense 5′-CCC​AGC​ATG​AGA​ACT​TCA​GG-3′ and anti-sense 5′-CAC​AGG​AAT​TCC​CAA​TGG​TC-3′; *CCND2*—sense 5′-GCG​GAG​AAG​CTG​TGC​ATT​TA-3′ and anti-sense 5′-CTG​CCA​GGT​TCC​ACT​TCA​AC-3′; *CDK4*—sense 5′-GTG​GAA​ACT​CTG​AAG​CCG​AC-3′, and anti-sense 5′-AAG​TCA​GCA​TTT​CCA​GCA​GC-3′; *mTOR*—sense 5′-ACC​TCA​CAA​GAC​ATC​GCT​GA-3′ and anti-sense 5′-CTC​TCT​CAC​CCA​GCA​GAA​CA-3′; *CTNNB1*—sense 5′-AAG​GTA​GAG​TGA​TGA​AAG​TTG​TT-3′ and anti-sense 5′-CAC​CAT​GTC​CTC​TGT​CTA​TTC-3′; and, *GAPDH*—sense 5′-CCT​GTT​CGA​CAG​TCA​GCC​G-3′ and anti-sense 5′-CGA​CCA​AAT​CCG​TTG​ACT​CC-3′. All primers were synthesized by Macrogen (Seoul, Korea). Independent-samples *t* test was used as statistical testing of gene expression comparing of standard-risk and high-risk MM subgroups.

### Western Blot Analysis

Cell lines were lyzed in radioimmunoprecipitation assay (RIPA) buffer (Cell Signaling Technology, Danvers, MA, United States) at 4°C for 5 min. The cell lysate was then centrifuged at 14,000*g* for 5 min and the supernatant was collected. Proteins were mixed with 1:1 Laemmli Sample Buffer (Bio-Rad Laboratories, Hercules, CA, United States), and then incubated at 95°C for 5 min. A slab of 10% SDS-polyacrylamide gel was prepared using a TGX FastCast™ Kit (Bio-Rad Laboratories). Proteins were separated by gel electrophoresis at 100 V for 1 h. After that, proteins were transferred to a membrane in transfer buffer (Bio-Rad Laboratories) at 100 V for 2 h, and then the membrane was blocked with blocking buffer that contained 5% non-fat dry milk in Tris-buffered saline with Tween 20 (TBST). The membrane was incubated with primary antibody UCHL1 (Cell Signaling Technology) and GAPDH (Abcam, Cambridge, United Kingdom) as an internal reference at room temperature for overnight and then incubated with secondary antibody anti-rabbit IgG, HRP-linked antibody at room temperature for 1 h (Cell Signaling Technology). The intensity of western blot results were performed by GeneTools software.

## Results

### Clinical and Laboratory Features of 15 MM Patients

**FIGURE 1:**
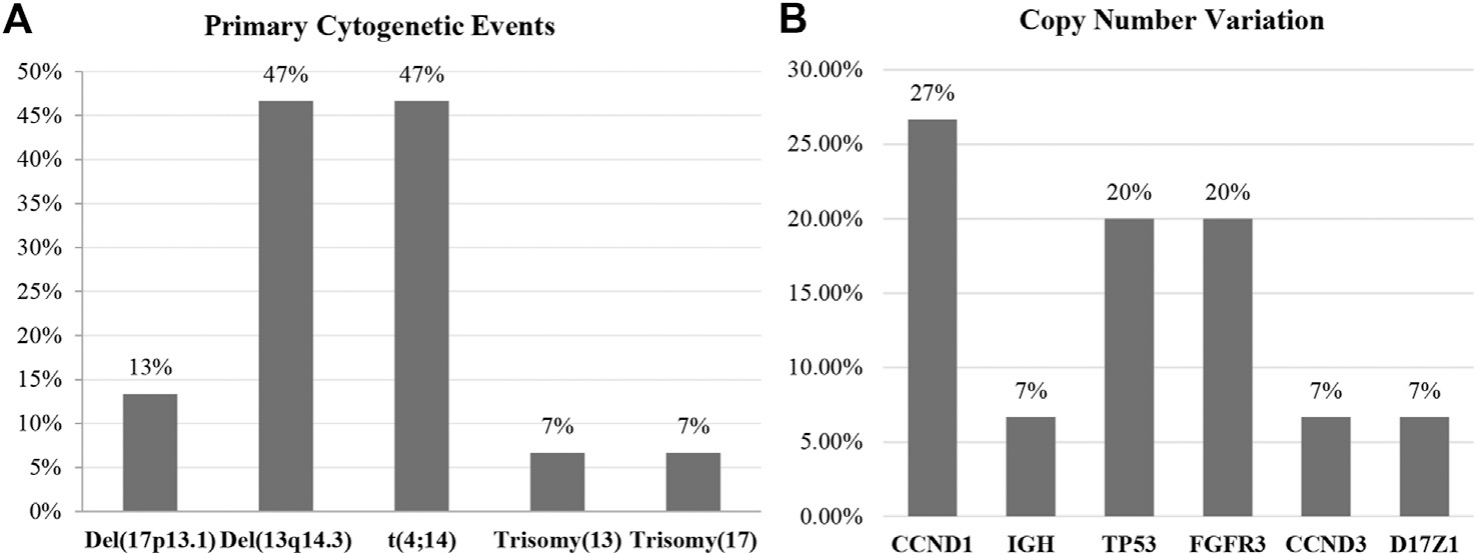
The overall genetic aberrations observed in this study using standard karyotyping and fluorescence *in situ* hybridization (FISH). **(A)** The most commonly observed primary cytogenetic event was del(13q14.3), and the high cytogenetic risk markers were t(4;14) and del(17p13.). **(B)** The secondary genetic event most frequently found in this study was copy number variation, with *CCND1* being most frequently observed followed by *TP53* and *FGFR3*.

We included 15 newly diagnosed MM patients in this study. Risk classification of patients was based on the IMWG consensus of risk stratification in MM [25]. There were 10 patients categorized as standard-risk, and five patients were allocated to the high-risk group. The clinical and laboratory features of the 15 included MM patients are presented in Table 1. Cytogenetic analysis by combination standard karyotyping and fluorescence *in situ* hybridization (FISH) revealed that t(4;14) and aberration on chromosome 13 were the most commonly observed cytogenetic abnormality in this study with a positivity rate among all cases of 47%. Four out of five cases in the high-risk group harbored a t(4;14). In addition, copy number gain of *CCDN1* was positive in 27% of all cases in this study while previous report about *CCDN1* copy number variation in Thai population was 15% [26]. Other genetic abnormalities observed in this study included copy number variation of *CCND1* (27%), *FGFR3* (20%), *TP53* (20%), *IGH* (7%), *CCND3* (7%), and *D17Z1* (7%). The overall genetic aberrations observed in this study are shown in Figure 1. Patient age ranged from 51 to 87 years, with a median of 65 years. The average values of β_2_-microglobulin, serum creatinine, and hemoglobin concentration are shown in Table 1. High-risk MM patients had a significantly higher β_2_-microglobulin value compared to standard-risk MM (*p* < 0.05). In addition, the median follow-up time of samples in the high-risk group was lower than the median follow-up time of all samples (30 months). Serum creatinine and hemoglobin concentrations were not significantly different between the high-risk group and standard-risk groups (Table 1).

### Differential Gene Expression in High-Risk Multiple Myeloma Versus Standard-Risk Multiple Myeloma

**FIGURE 2:**
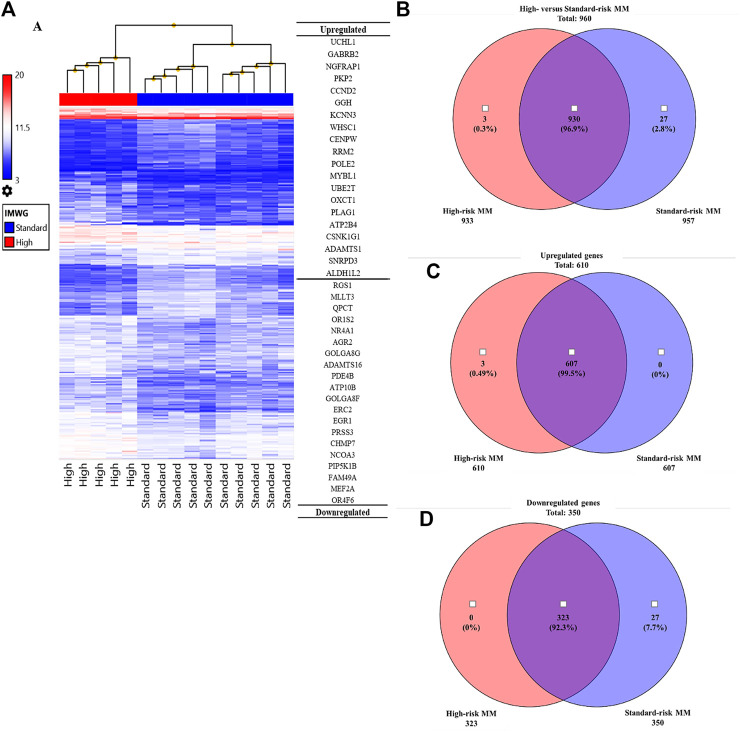
**(A)** Hierarchical clustering of the high cytogenetic risk group compared to the standard cytogenetic risk group. The samples that had the most similar gene expression patterns were grouped and connected by a series of branches. Even though the expression patterns of the high and standard cytogenetic risk groups were for the most part similar, the gene expression patterns of high cytogenetic risk samples (red bar) were different from those of standard cytogenetic risk samples at the first-line branch. The top 20 upregulated and downregulated genes followed by fold change are shown. The highest fold change of an upregulated gene in the high cytogenetic risk group was *UCHL1*. **(B)** Venn diagram of total genes from the high cytogenetic risk group and the standard cytogenetic risk group. Most of the differentially expressed genes were found in both the high and standard cytogenetic risk groups (930 genes, 96.9%). **(C)** Venn diagram of upregulated genes. Most upregulated genes were found in both cytogenetic risk groups, but there were three genes only found in the high cytogenetic risk group, and all three of them were upregulated genes. No upregulated genes were found only in the standard cytogenetic risk group. **(D)** Venn diagram of downregulated genes. Most downregulated genes were also found in both cytogenetic risk groups. This result indicates that the transcriptome molecular signatures of high and standard cytogenetic risk MM are very close in pattern of expression.

To identify genes and pathways that were differentially expressed in the high-risk group compared with the standard-risk group, we performed gene expression profiling using Clariom™ S Array GeneChip from Affymetrix, and we applied ANOVA to filter the differentially expressed genes using TAC software. As expected, the expression profiles of high-risk and standard-risk MM exhibited a high degree of difference in gene transcription levels. The hierarchical plot that is shown in Figure 2A clearly demonstrates the differences in cytogenetic risk between groups. The top 20 upregulated and downregulated genes compared between the high-risk and standard-risk groups are shown in Figure 2A. Interestingly, the highest differentially overexpressed gene is *UCHL1* and we found *CCND2*, one of its downstream target, also overexpressed in the top 20 upregulated genes. Details specific to the top 50 upregulated and downregulated genes are shown in Supplementary Tables S1 and S2, respectively. There were 960 genes differentially expressed between the high-risk and standard-risk groups with a fold change greater than the 2.0 cutoff (*p* < 0.05) (Figure 2B). Among those 960 signatures, there were 610 genes (63.54%) found to be overexpressed (fold change >2.0), as shown by Venn diagram comparing the high-risk group with the standard-risk group in Figure 2C. Interestingly, several upregulated genes in high-risk MM were commonly involved in cell cycle control, the Wnt signaling pathway, and the ubiquitin proteasome pathway. In contrast, there were 350 genes (36.46%) found to be significantly downregulated (fold change <2.0, *p* < 0.05), as shown by Venn diagram comparing the high-risk group with the standard-risk group in Figure 2D. Collectively, these findings further highlight the difference in the transcriptional signatures of these two cytogenetic risk subgroups of MM. Additionally, dysregulation of a key component of the proteasome pathway, *UCHL1* might contribute to high-risk transcriptional signature by disrupting several components of cell cycle control, including *CCND2*.

### Gene Set Enrichment Analysis and Pathway Analysis of Genes Differentially Expressed in High-Risk Multiple Myeloma

**FIGURE 3:**
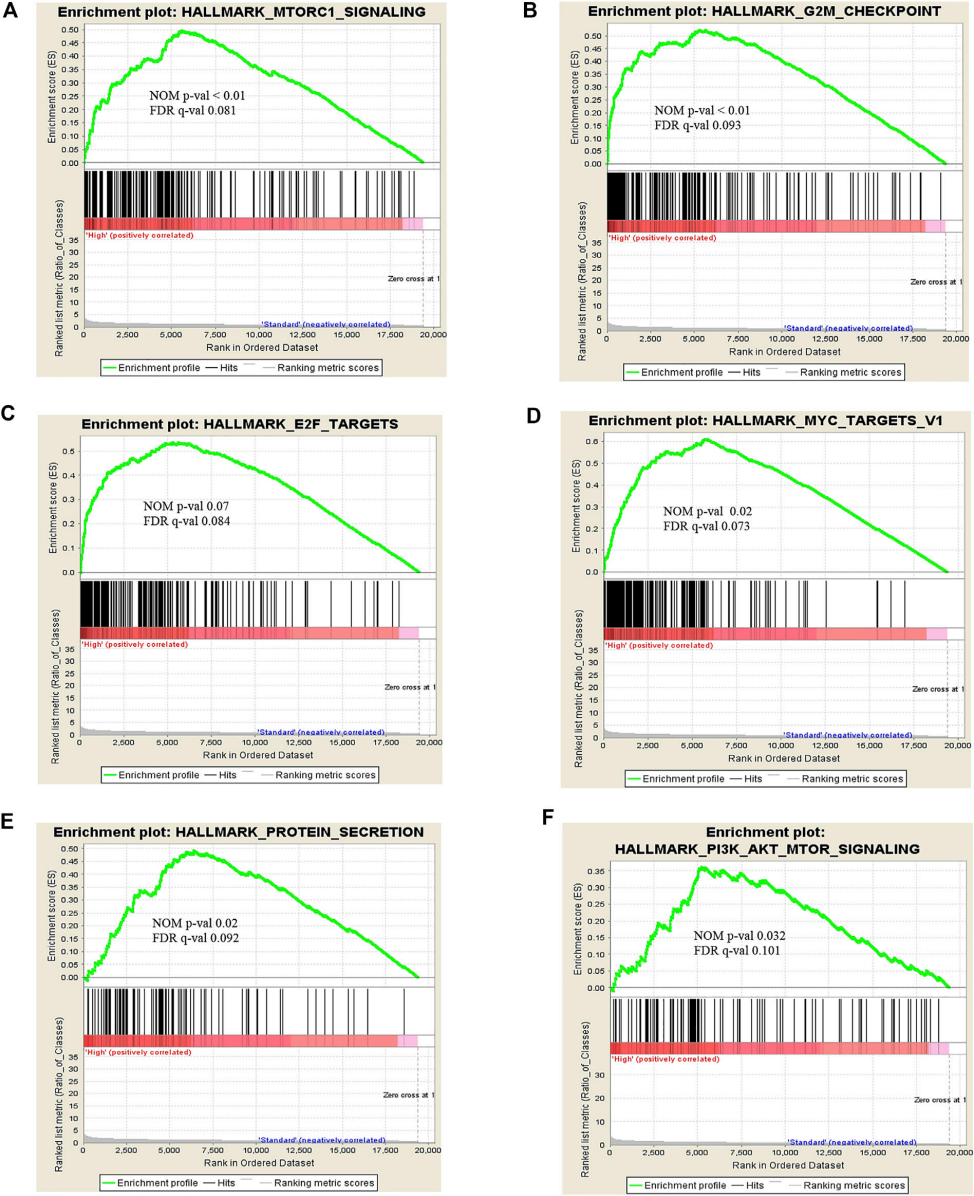
Snapshot of enrichment results from Gene Set Enrichment Analysis (GSEA) shows the gene sets that were enriched in the t(4;14) group compared to the non-t(4;14) group. The enriched gene sets are mostly involved in cell cycle control and protein metabolism, including **(A)** MTORC1 signaling, **(B)** G2M checkpoint, **(C)** E2F targets, **(D)** MYC targets V1, **(E)** Protein secretion, and **(F)** PI3K-AKT-MTOR signaling.

**FIGURE 4:**
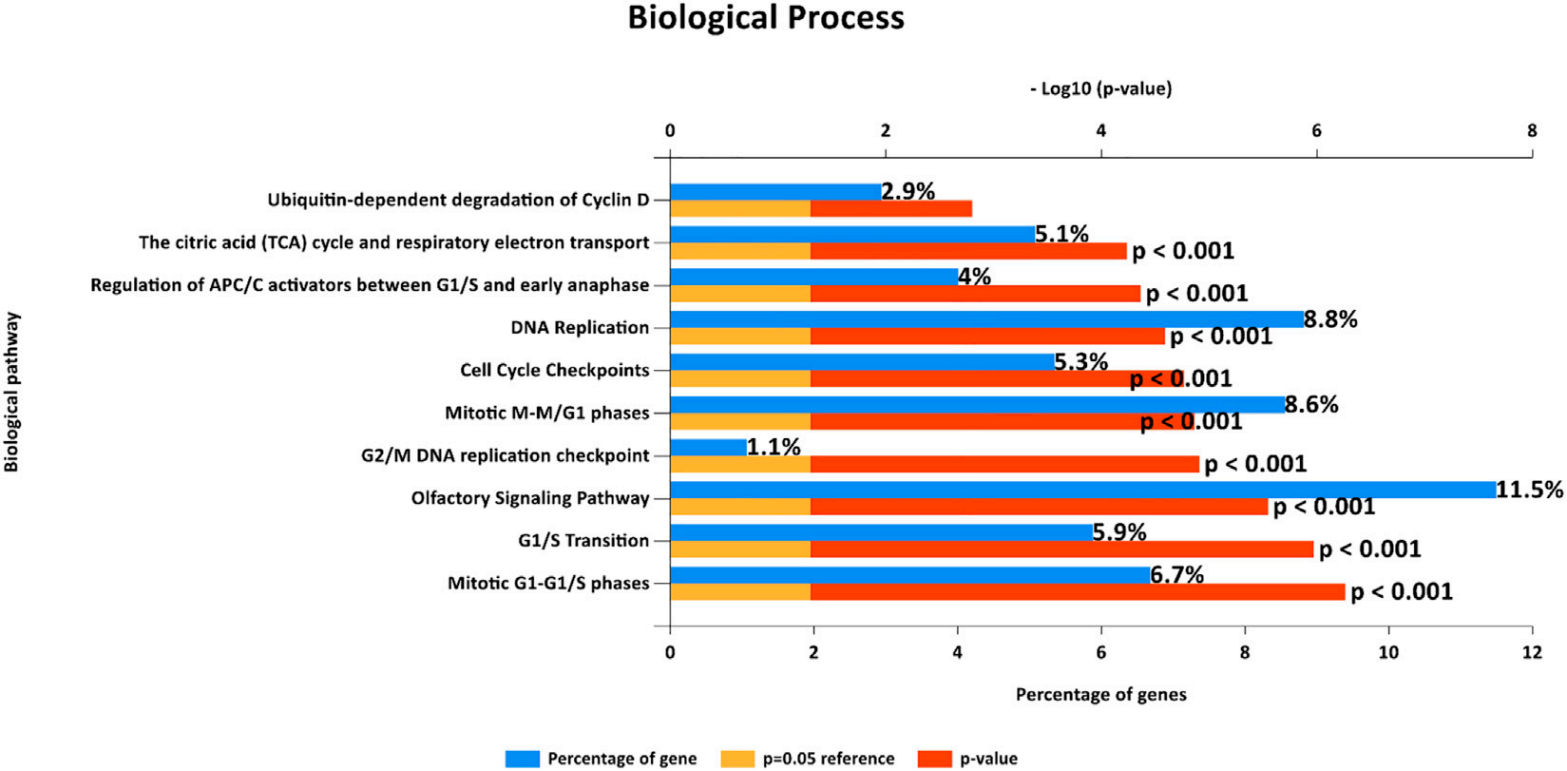
Biological pathways from FunRich software demonstrate enriched gene sets that show the same enrichment results as those observed from Gene Set Enrichment Analysis (GSEA) in terms of cell cycle control.

**FIGURE 5:**
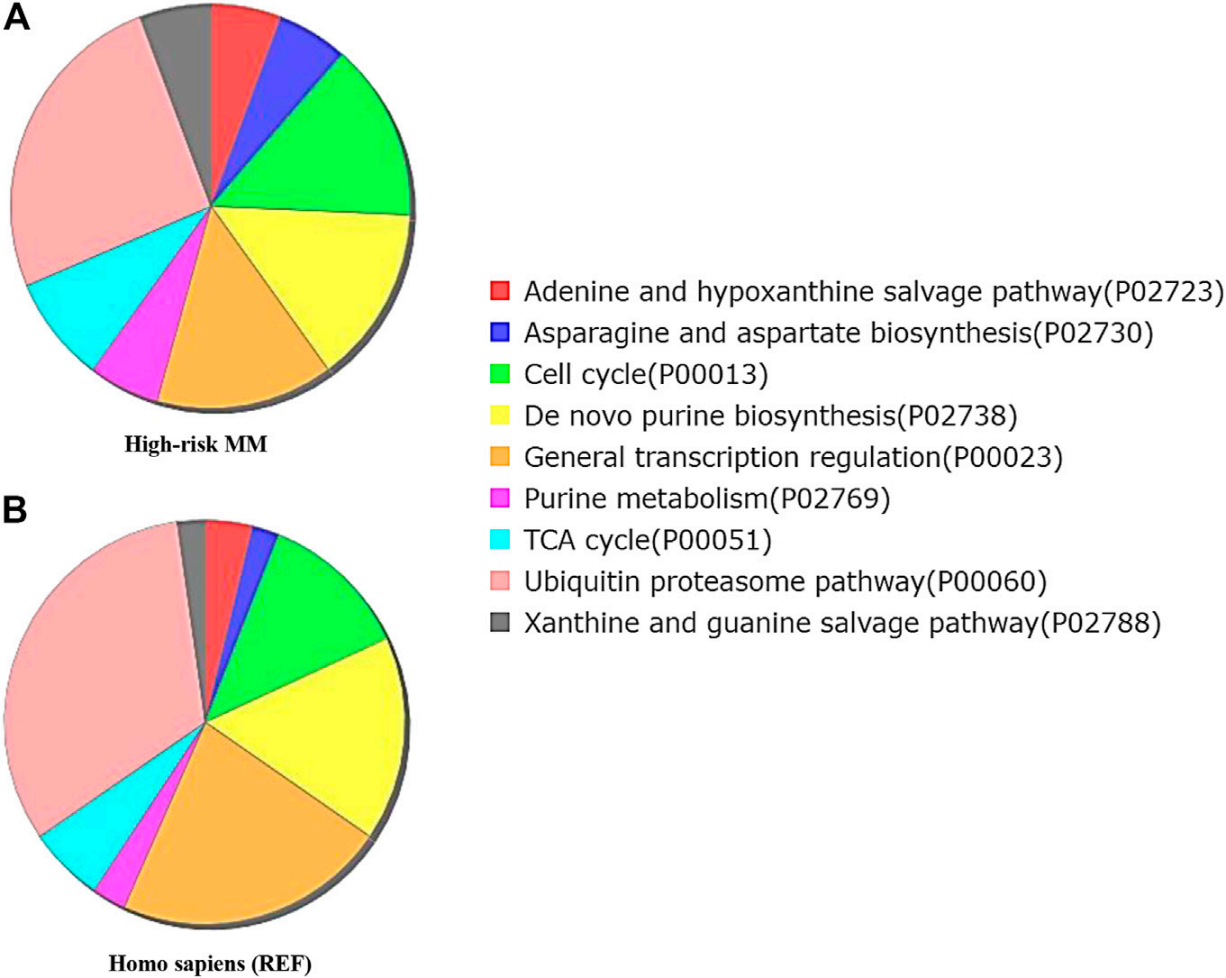
Functional enrichment analysis by Gene Ontology (GO) Panther pathway demonstrates a pie chart of the t(4;14) MM group **(A)** compared to reference Homo sapiens **(B)**. Gene sets involved in the cell cycle were more frequently found in the high cytogenetic risk group (green color). Interestingly, the ubiquitin proteasome pathway was found less frequently in the high cytogenetic risk group than in reference Homo sapiens. This finding combined with the observed high fold change of *UCHL1* from expression profile analysis suggests the involvement of *UCHL1* in multiple functions, including the ubiquitin proteasome degradation system and cell cycle control.

To explore the certain biological process or molecular function of differentially expressed genes, we performed three different classes of pathway analysis, including Gene Set Enrichment Analysis (GSEA), FUNRICH, and Gene Ontology (GO) Panther pathway enrichment analysis, to analyze our data. GSEA revealed that 16 gene sets were significantly enriched at a nominal *p*-value < 5% and a false discovery rate (FDR) < 25%, as shown in Supplementary Table S3. These included hallmark of mTOR signaling, G2M cell cycle checkpoint, E2F targets, MYC targets, and protein secretion pathways (Figure 3). Similar to GSEA, FUNRICH pathway analysis showed several pathways associated with tumorigenesis and tumor progression to be enriched. Those included Wnt signaling, cell cycle control, G2M cell cycle checkpoint, ubiquitin-dependent degradation of cyclin D, mTORC1 signaling, PI3K-AKT-mTOR signaling, and DNA repair pathways (Figure 4). Consistent to GSEA and FUNRICH, GO enrichment analysis further confirmed cell cycle control and the ubiquitin proteasome pathway to be enriched in the high-risk MM group (Figure 5). Taken together, our data indicate the disruption of several molecular signaling pathways in genes differentially expressed among t(4;14) MM and non-t(4;14) MM. Additionally, these data further highlight the alteration of the ubiquitin proteasome pathway, and they suggest that *UCHL1* influences tumorigenesis and tumor progression among t(4;14) MM.

### UCHL1 and Its Downstream Target CCDN2 Were Overexpressed in Cytogenetic High-Risk MM

**FIGURE 6:**
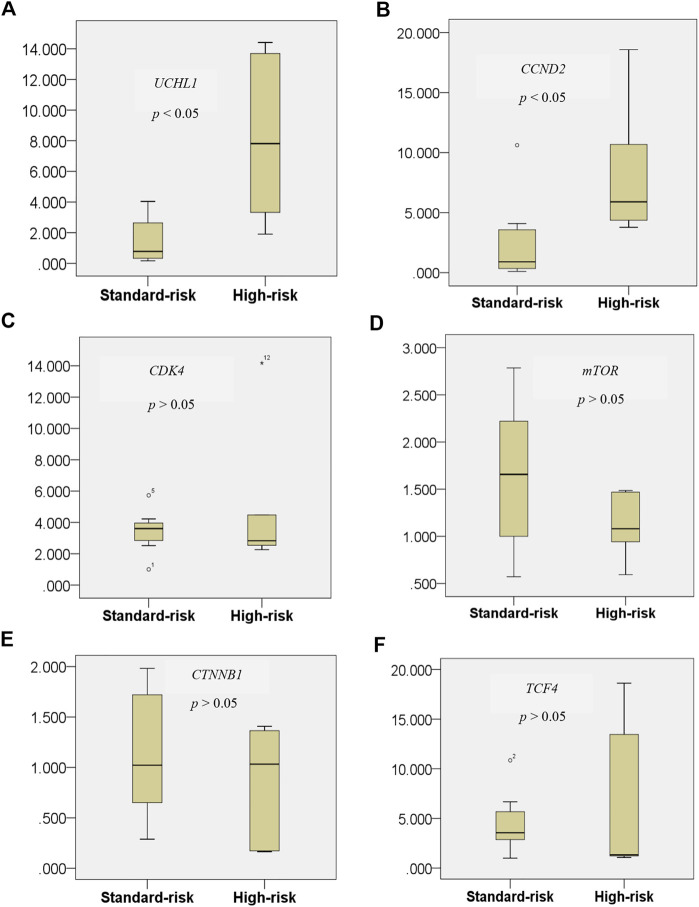
Expression level of the *UCHL1* gene in the t(4;14) and non-t(4;14) MM groups **(A)**, and of the *UCHL1* downstream target genes *CCND2* **(B)**, *CDK4* **(C)**, *mTOR* **(D)**, *CTNNB1* **(E)**, and *TCF4* **(F)**. UCHL1 mRNA expression was found to be significantly higher in the high cytogenetic risk group than in the standard cytogenetic risk group. Among all of the well-known downstream targets of *UCHL1* from previous studies, only *CCND2* was found to be significantly differentially expressed in the t(4;14) MM group. These data further highlight the copersistence of upregulation of *UCHL1* and *CCND2* in t(4;14) high cytogenetic risk MM, which may be involved in disease progression in this MM subgroup.

Gene set enrichment and pathway analysis data both demonstrated that the ubiquitin proteasome pathway via *UCHL1* and *CCND2*-dependent cell cycle control pathways are copersistent and differentially expressed in t(4;14) high cytogenetic risk MM compared to the other non-t(4;14) MM group. It was earlier reported that *UCHL1* regulates cell cycle control and enhances cell proliferation by activation of several cyclin-dependent kinase proteins [21]. Perhaps more importantly, previous study found that *UCHL1* promotes progression of colorectal cancer cell lines via the activation of *CCND2*, which is a downstream target of the Wnt β-catenin/TCF pathway [18]. Furthermore, *UCHL1* activates the Akt pathways to promote the progression of osteosarcoma [27] and breast cancer [28]. These findings prompted us to further investigate whether key components of those pathways were differentially expressed in our tested samples. By RT-qPCR, *UCHL1* and its downstream target *CCDN2* were significantly differentially overexpressed in the high cytogenetic risk group (*p* < 0.05) (Figure 6). Our data further highlight the copersistence of upregulation of *UCHL1* and *CCND2* in t(4;14) high cytogenetic risk MM, which may be involved in disease progression in this MM subgroup.

### Targeting UCHL1 Could Arrest Cytogenetic High-Risk Multiple Myeloma Cell Lines

**FIGURE 7:**
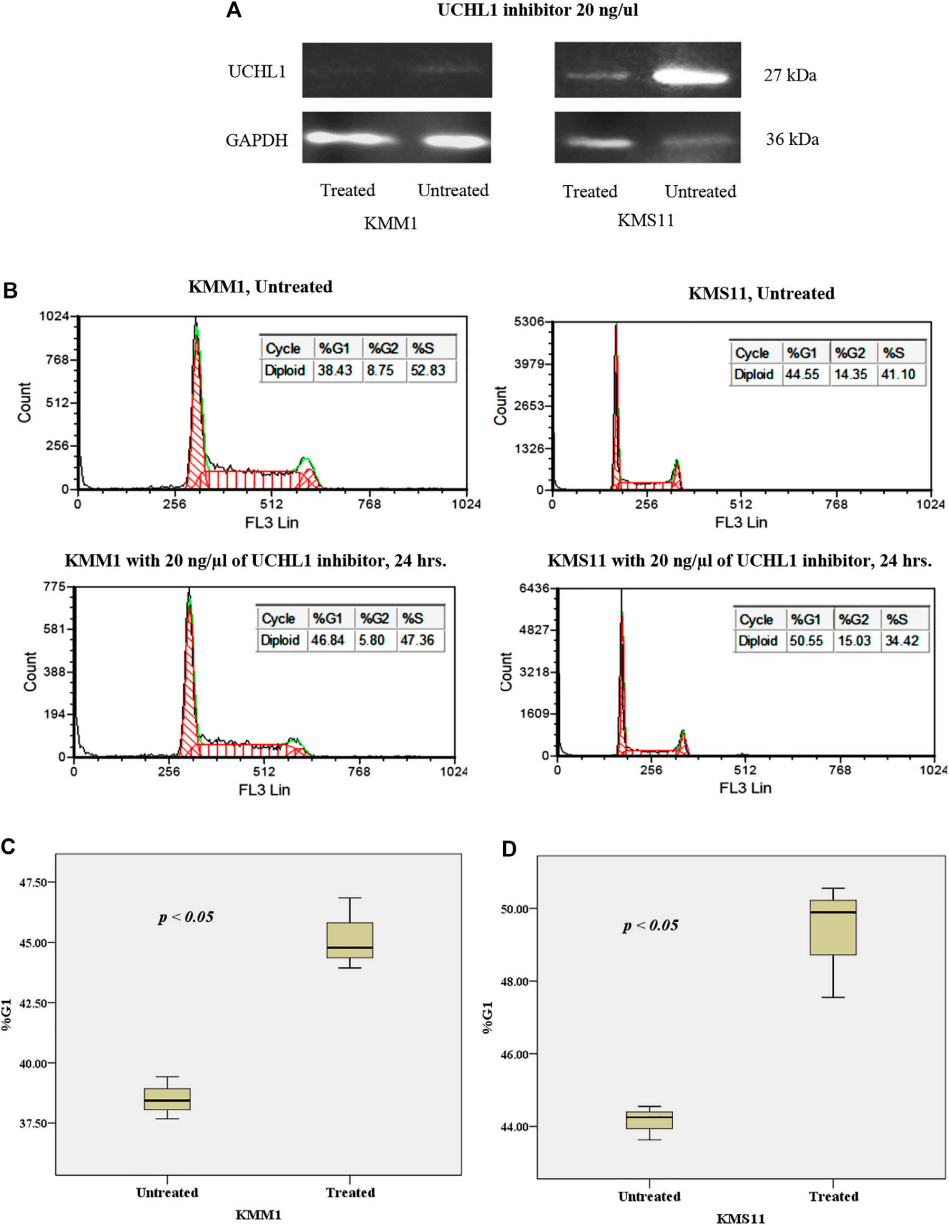
**(A)** Western blot analysis of the effect of UCHL1 inhibitor on UCHL1 protein level in KMM1 and KMS11 cell lines after treatment. UCHL1 inhibitor could suppress the protein level of UCHL1 in KMS11 cell line, which is a phenotypically overexpressed UCH1 protein. **(B)** DNA index of KMM1 and KMS11 in both treated and untreated conditions. After treatment with 20 ng/μl of UCHL1 inhibitor for 24 h, the DNA index demonstrated an increase in the %G1 phase in the treated condition. Results shown the significantly increased of %G1 phase both **(C)** KMM1 and **(D)** KMS11 cell lines. These results indicate that UCHL1 can manipulate cell cycle control in MM cell line in both high and low expression of UCHL1 protein.

To gain further insights into the mechanism of UCHL1 that is responsible for the progression of t(4;14) cytogenetic high-risk MM, we performed *in vitro* pharmacological targeting of UCHL1 in high-risk with t(4;14) (KMS11) and non-t(4;14) (KMM1) cell lines using UCHL1 inhibitor (LDN-57444). LDN-57444 has been used to inhibit UCHL1 hydrolase activity leading to cell death via apoptosis by decreasing the activity of ubiquitin proteasome [29]. Both KMS11 and KMM1 were treated with 20 ng/μl of LDN-57444 for 24 h. Cell proliferation results established by using tryphan blue staining showed significantly decreased cell numbers in KMM1 and KMS11 cell lines (*p*-value < 0.05) as shown in Supplementary Figure S1. The efficacy of LDN-57444 to target UCHL1 was confirmed Western blotting. As expected, targeting of UCHL1 was able to suppress the protein level and proliferation of both KMS11 and KMM1 cell lines *in vitro*. Interestingly, KMS11 cells with phenotypically overexpressed UCHL1 protein were strongly suppressed by LDN-57444 compared to KMM1 cells with a low level of UCHL1 protein (Figure 7A). The intensity of western blot results of treated and untreated conditions in KMM1 were 6.85 × 10^4^ and 1.62 × 10^5^ respectively, whereas in KMS11 were 3.8 × 10^5^ and 3.44 × 10^6^, respectively. Additionally, cell cycle arrest in G1 was observed in both KMS11 and KMM1 cell lines after treatment with UCHL1 inhibitor (Figure 7B). Statistical analysis demonstrated that both cell lines had significantly increased proportion in G1 phase after treatment (Figures 7C,D). These results suggest that both cell lines were sensitized by UCHL1 inhibitor resulting in increased cell cycle proportion in G1 phase even though in KMS11 cells with phenotypic overexpression of UCHL1. We further investigated the crosstalk between UCHL1 and its downstream targets, including CCND2, CDK4, mTOR, and β-catenin. Using STRING database to predict protein-protein interaction (PPI). We found that UCHL1 is able to interact with CCND2 via CDKN1B and β-catenin (Supplementary Figure S2A). Another PPI network was generated using UCHL1 and the top 100 upregulated genes identified in the expression profile analysis in this study. The result showed that UCHL1 can interact with the molecular network involved with CCND2 by indirect interaction (Supplementary Figure S2B). Moreover, functional enrichment in the predicted protein network was mostly observed in proteins that influence cell cycle regulatory pathways, which is similar to our findings from functional enrichment analysis (Supplementary Tables S3–S5). Taken together, our data further highlight the potential molecular mechanism of UCHL1 to manipulate the cell cycle in MM cell lines.

## Discussion

MM is known as an incurable disease that is very heterogeneous in clinical presentation, genetic alteration, treatment response, and disease survival, especially in patients in the high cytogenetic risk group. Several cytogenetic abnormalities were recently identified as high-risk markers in MM patients, including t(4;14), t(14;16), t(14;20), del(17/17p), non-hyperdiploidy of chromosome number, and gain (1q) according to IMWG risk classification 2016 [22]. This is similar to previous studies which reported that translocation involving immunoglobulin heavy chain, including t(4;14), is a common cytogenetic abnormality in high-risk MM patients [10, 22, 30, 31]. Furthermore, t(4;14) was associated with an adverse prognosis in patients who received high-dose chemotherapy with autologous stem cell transplantation (ASCT) [32–34]. Expression profile and functional enrichment analyses yield information about the molecular machinery in MM cells, such as the immune system [35], cell cycle regulation [36, 37], and cell differentiation and proliferation [38], which can lead to the identification of promising biomarkers and potential therapeutic targets for the treatment of MM.

In this work, using high-resolution gene expression profiling analysis, we found that 96.9% of genes have called as intersect part of both the high-risk and standard-risk groups of MM from Venn diagram but the level of gene expression pattern between these groups have been significantly difference as the result shown in Hierarchical clustering (Figure 2). This highlights the complicated nature of the molecular machinery of this disease, and the difficulty associated with identifying a unique biomarker in both MM subtypes. Four of five samples in the high-risk group harbored t(4;14). This translocation was frequently found to be involved in the alteration of the FGFR3 and WHSC1 genes due to their juxtaposition next to IGH@ enhancers [39, 40]. Interestingly, we found *UCHL1* to be predominantly differentially overexpressed in those samples when compared with samples derived from standard-risk MM patients and high-risk MM patients not harboring t(4;14). Several studies reported that *UCHL1* is a key regulator of the invasion and metastasis of several tumors [41], including pancreatic neuroendocrine tumors [42], pediatric high-grade glioma [43], breast carcinoma [44], and ovarian cancer [45]. *UCHL1* was also reported to be a biomarker for t(4;14) aggressive MM [11]. In mouse model, *UCHL1* was found to be crucial for the development of B-cell lineage, which shows as plasmacytoma histology [46]. However, it is less clear regarding the molecular mechanism of *UCHL1* and its downstream targets in the establishment of MM. To address this, we investigated the molecular functions of *UCHL1* using expression microarray and newly developed functional enrichment analysis tools. Similar to previous functional studies in the cell cycle, we found several genes and gene set data from enrichment analysis that indicated that *UCHL1* is required for cell cycle process and cell survival [11, 20, 47, 48]. Our data further confirmed that *UCHL1* expression level is significantly overexpressed in high-risk MM patients with t(4;14).

Surprisingly, of all of the well-defined *UCHL1* downstream targets, including *CCND2*, *mTOR*, *CDK4*, *CTNNB1*, and *TCF4*, only *CCND2* was found to be significantly overexpressed commensurate with *UCHL1* expression level. This result supports the previously reported finding that high expression of *UCHL1* correlated with D type cyclin [49, 50].

We performed additional experiments to address the hypothesis that small molecular inhibitor could manipulate t(4;14) MM cells via UCHL1 inhibition. Our results revealed that UCHL1 inhibitor could suppress the UCHL1 protein level of MM cell line when compared with control condition. This observation may be explained by the fact that small molecule inhibitor can target the UCHL1 protein at specific active-site structure [51]. Moreover, this result related to DNA index, which suggests that UCHL1 inhibition leads to cell cycle arrest in G1 phase. This data further suggests the potential impact of targeting UCHL1 on cell survival in MM cell lines. Moreover, our data from Western blot analysis further confirmed that UCHL1 protein level is overexpressed in KMS11 cell line that is positive for t(4;14), and 4 out of 5 of our tested samples in the high-risk group harbored t(4;14). This data further supports previous reports of *UCHL1* high expression in MM cells with t(4;14) [11, 46]. We then generated a protein-protein interaction network, which revealed that UCHL1 is involved in the regulation of cell cycle process via cyclin D. However, additional study is needed to further elucidate the molecular function of *UCHL1* in the development of MM, such as genetic suppression of *UCHL1* in MM cell lines and in *vivo* mouse model.

## Conclusion

Our data demonstrate the differential gene expression patterns in the high-risk and standard-risk groups of MM. *UCHL1* was found to be predominantly overexpressed in the t(4;14) high cytogenetic risk group, which had an 80% prevalence of t(4;14) positivity. Gene set enrichment and pathway analysis revealed that *UCHL1* regulates cell cycle control in t(4;14) high-risk MM. Additionally, data from the protein-protein interaction network generated and the *in vitro* experiments conducted in this study suggest that *UCHL1* regulates the cell cycle in MM cells. Finally, targeting of UCHL1 using LDN-57444 could suppress the growth of t(4;14) high-risk MM cell lines *in vitro* to arrest the cell cycle in G1 phase. Future works will aim to investigate the genetic suppression of *UCHL1* in both *in vitro* cell line and in mouse model, and to study the functional crosstalk of *UCHL1* and its downstream targets, such as *CCND2* and β-catenin, in the development of MM.

## Data Availability

The original contributions presented in the study are included in the article/Supplementary Material, further inquiries can be directed to the corresponding author.

## References

[1] Palumbo A Anderson K. Multiple myeloma. N Engl J Med (2011) 364(11):1046–60. 10.1056/NEJMra1011442 21410373

[2] Committee of The Ramathibodi Hospital Cancer Center. Ramathibodi cancer report 2017. Bangkok, Thailand: Ramathibodi Comprehensive Cancer Center, Faculty of Medicine, Ramathibodi Hospital, Mahidol University (2017).

[3] Bazarbachi AH Al Hamed R Malard F Harousseau JL Mohty M. Relapsed refractory multiple myeloma: a comprehensive overview. Leukemia (2019) 33(10):2343–57. 10.1038/s41375-019-0561-2 31455853

[4] Bittner M Meltzer P Chen Y Jiang Y Seftor E Hendrix M Molecular classification of cutaneous malignant melanoma by gene expression profiling. Nature (2000) 406(6795):536–40. 10.1038/35020115 10952317

[5] Zhan F Hardin J Kordsmeier B Bumm K Zheng M Tian E Global gene expression profiling of multiple myeloma, monoclonal gammopathy of undetermined significance, and normal bone marrow plasma cells. Blood (2002) 99(5):1745–57. 10.1182/blood.v99.5.1745 11861292

[6] Meissner T Seckinger A Reme T Hielscher T Mohler T Neben K Gene expression profiling in multiple myeloma–reporting of entities, risk, and targets in clinical routine. Clin Cancer Res (2011) 17(23):7240–7. 10.1158/1078-0432.Ccr-11-1628 21986844

[7] Weinhold N Heuck CJ Rosenthal A Thanendrarajan S Stein CK Van Rhee F Clinical value of molecular subtyping multiple myeloma using gene expression profiling. Leukemia (2016) 30(2):423–30. 10.1038/leu.2015.309 26526987 PMC4740265

[8] Lohr JG Stojanov P Carter SL Cruz-Gordillo P Lawrence MS Auclair D Widespread genetic heterogeneity in multiple myeloma: implications for targeted therapy. Cancer Cell (2014) 25(1):91–101. 10.1016/j.ccr.2013.12.015 24434212 PMC4241387

[9] Chng WJ Chung TH Kumar S Usmani S Munshi N Avet-Loiseau H Gene signature combinations improve prognostic stratification of multiple myeloma patients. Leukemia (2016) 30(5):1071–8. 10.1038/leu.2015.341 26669975

[10] Morgan GJ Walker BA Davies FE. The genetic architecture of multiple myeloma. Nat Rev Cancer (2012) 12(5):335–48. 10.1038/nrc3257 22495321

[11] Hussain S Bedekovics T Chesi M Bergsagel PL Galardy PJ. UCHL1 is a biomarker of aggressive multiple myeloma required for disease progression. Oncotarget (2015) 6(38):40704–18. 10.18632/oncotarget.5727 26513019 PMC4747363

[12] Larsen CN Price JS Wilkinson KD. Substrate binding and catalysis by ubiquitin C-terminal Hydrolases: identification of two active site residues. Biochemistry (1996) 35(21):6735–44. 10.1021/bi960099f 8639624

[13] Setsuie R Wada K. The functions of UCH-L1 and its relation to neurodegenerative diseases. Neurochem Int (2007) 51(2-4):105–11. 10.1016/j.neuint.2007.05.007 17586089

[14] Andersson FI Werrell EF McMorran L Crone WJ Das C Hsu ST The effect of Parkinson's-disease-associated mutations on the deubiquitinating enzyme UCH-L1. J Mol Biol (2011) 407(2):261–72. 10.1016/j.jmb.2010.12.029 21251915

[15] Mani A Gelmann EP. The ubiquitin-proteasome pathway and its role in cancer. J Clin Oncol (2005) 23(21):4776–89. 10.1200/JCO.2005.05.081 16034054

[16] Hussain S Zhang Y Galardy PJ. DUBs and cancer: the role of deubiquitinating enzymes as oncogenes, non-oncogenes and tumor suppressors. Cell Cycle (2009) 8(11):1688–97. 10.4161/cc.8.11.8739 19448430

[17] Bedekovics T Hussain S Feldman AL Galardy PJ. UCH-L1 is induced in germinal center B cells and identifies patients with aggressive germinal center diffuse large B-cell lymphoma. Blood (2016) 127(12):1564–74. 10.1182/blood-2015-07-656678 26702068 PMC4807423

[18] Zhong J Zhao M Ma Y Luo Q Liu J Wang J UCHL1 acts as a colorectal cancer oncogene via activation of the beta-catenin/TCF pathway through its deubiquitinating activity. Int J Mol Med (2012) 30(2):430–6. 10.3892/ijmm.2012.1012 22641175

[19] Goto Y Zeng L Yeom CJ Zhu Y Morinibu A Shinomiya K UCHL1 provides diagnostic and antimetastatic strategies due to its deubiquitinating effect on HIF-1alpha. Nat Commun (2015) 6:6153. 10.1038/ncomms7153 25615526 PMC4317501

[20] Hussain S Bedekovics T Liu Q Hu W Jeon H Johnson SH UCH-L1 bypasses mTOR to promote protein biosynthesis and is required for MYC-driven lymphomagenesis in mice. Blood (2018) 132(24):2564–74. 10.1182/blood-2018-05-848515 30257881 PMC6293873

[21] Kabuta T Mitsui T Takahashi M Fujiwara Y Kabuta C Konya C Ubiquitin C-terminal hydrolase L1 (UCH-L1) acts as a novel potentiator of cyclin-dependent kinases to enhance cell proliferation independently of its hydrolase activity. J Biol Chem (2013) 288(18):12615–26. 10.1074/jbc.M112.435701 23543736 PMC3642309

[22] Sonneveld P Avet-Loiseau H Lonial S Usmani S Siegel D Anderson KC Treatment of multiple myeloma with high-risk cytogenetics: a consensus of the International Myeloma Working Group. Blood (2016) 127(24):2955–62. 10.1182/blood-2016-01-631200 27002115 PMC4920674

[23] Szklarczyk D Gable AL Lyon D Junge A Wyder S Huerta-Cepas J STRING v11: protein–protein association networks with increased coverage, supporting functional discovery in genome-wide experimental datasets. Nucleic Acids Res (2018) 47(D1):D607–D613. 10.1093/nar/gky1131 PMC6323986 30476243

[24] Gong B Cao Z Zheng P Vitolo OV Liu S Staniszewski A Ubiquitin hydrolase uch-L1 rescues β-amyloid-induced decreases in synaptic function and contextual memory. Cell (2006) 126(4):775–88. 10.1016/j.cell.2006.06.046 16923396

[25] Chng WJ Dispenzieri A Chim CS Fonseca R Goldschmidt H Lentzsch S IMWG consensus on risk stratification in multiple myeloma. Leukemia (2014) 28(2):269–77. 10.1038/leu.2013.247 23974982

[26] Phornsarayuth P KorKiasakul V Puavilai T Boonyawat K Niparuck P Chuncharunee S Molecular cytogenetic aberrations in Thai patients with multiple myeloma. Genomics Genet (2016) 9:25–30. 10.14456/gag.2016.4

[27] Zheng S Qiao G Min D Zhang Z Lin F Yang Q Heterogeneous expression and biological function of ubiquitin carboxy-terminal hydrolase-L1 in osteosarcoma. Cancer Lett (2015) 359(1):36–46. 10.1016/j.canlet.2014.12.001 25578779

[28] Luo Y He J Yang C Orange M Ren X Blair N UCH-L1 promotes invasion of breast cancer cells through activating Akt signaling pathway. J Cell Biochem (2018) 119(1):691–700. 10.1002/jcb.26232 28636190 PMC5705264

[29] Tan YY Zhou HY Wang ZQ Chen SD. Endoplasmic reticulum stress contributes to the cell death induced by UCH-L1 inhibitor. Mol Cell Biochem (2008) 318(1-2):109–15. 10.1007/s11010-008-9862-x 18622688

[30] Hu Y Chen W Chen S Huang Z. Cytogenetic abnormality in patients with multiple myeloma analyzed by fluorescent *in situ* hybridization. Onco Targets Ther (2016) 9:1145–9. 10.2147/OTT.S95818 27042105 PMC4780433

[31] Yoshida T Ri M Fujinami H Oshima Y Tachita T Marumo Y Impact of chromosomal abnormalities on the efficacy of lenalidomide plus dexamethasone treatment in patients with relapsed/refractory multiple myeloma. Int J Hematol (2019) 110(2):228–36. 10.1007/s12185-019-02669-z 31119611

[32] Sahota SS Leo R Hamblin TJ Stevenson FK. Ig VH gene mutational patterns indicate different tumor cell status in human myeloma and monoclonal gammopathy of undetermined significance. Blood (1996) 87(2):746–55. 10.1182/blood.V87.2.746.bloodjournal872746 8555499

[33] Walker BA Leone PE Jenner MW Li C Gonzalez D Johnson DC Integration of global SNP-based mapping and expression arrays reveals key regions, mechanisms, and genes important in the pathogenesis of multiple myeloma. Blood (2006) 108(5):1733–43. 10.1182/blood-2006-02-005496 16705090

[34] Anderson K Lutz C van Delft FW Bateman CM Guo Y Colman SM Genetic variegation of clonal architecture and propagating cells in leukaemia. Nature (2011) 469(7330):356–61. 10.1038/nature09650 21160474

[35] Sun J Wen X Jin F Li Y Hu J Sun Y. Bioinformatics analyses of differentially expressed genes associated with bisphosphonate-related osteonecrosis of the jaw in patients with multiple myeloma. OncoTargets Ther (2015) 8:2681–8. 10.2147/OTT.S88463 PMC4590669 26445550

[36] David CJ Massagué J. Contextual determinants of TGFβ action in development, immunity and cancer. Nat Rev Mol Cel Biol (2018) 19(7):419–35. 10.1038/s41580-018-0007-0 PMC7457231 29643418

[37] Chattopadhyay S Thomsen H Yadav P da Silva Filho MI Weinhold N Nöthen MM Genome-wide interaction and pathway-based identification of key regulators in multiple myeloma. Commun Biol (2019) 2(1):89. 10.1038/s42003-019-0329-2 30854481 PMC6399257

[38] Yan H Zheng G Qu J Liu Y Huang X Zhang E Identification of key candidate genes and pathways in multiple myeloma by integrated bioinformatics analysis: YAN et al. J Cell Physiol (2019) 234(12):23785–97. 10.1002/jcp.28947 31215027 PMC6771956

[39] Akiyama M Hideshima T Shammas MA Hayashi T Hamasaki M Tai YT Effects of oligonucleotide N3′→P5′ thio-phosphoramidate (GRN163) targeting telomerase RNA in human multiple myeloma cells. Cancer Res (2003) 63(19):6187–94. 14559802

[40] Walker BA Wardell CP Melchor L Hulkki S Potter NE Johnson DC Intraclonal heterogeneity and distinct molecular mechanisms characterize the development of t(4;14) and t(11;14) myeloma. Blood (2012) 120(5):1077–86. 10.1182/blood-2012-03-412981 22573403

[41] Kim HJ Kim YM Lim S Nam YK Jeong J Kim HJ Ubiquitin C-terminal hydrolase-L1 is a key regulator of tumor cell invasion and metastasis. Oncogene (2009) 28(1):117–27. 10.1038/onc.2008.364 18820707

[42] Moore MD Finnerty B Gray KD Hoda R Liu YF Soong L Decreased UCHL1 expression as a cytologic biomarker for aggressive behavior in pancreatic neuroendocrine tumors. Surgery (2018) 163(1):226–31. 10.1016/j.surg.2017.04.040 29150024

[43] Sanchez-Diaz PC Chang JC Moses ES Dao T Chen Y Hung JY. Ubiquitin carboxyl-terminal esterase L1 (UCHL1) is associated with stem-like cancer cell functions in pediatric high-grade glioma. PLoS One (2017) 12(5):e0176879. 10.1371/journal.pone.0176879 28472177 PMC5417601

[44] Lien HC Wang CC Lin CH Lu YS Huang CS Hsiao LP Differential expression of ubiquitin carboxy-terminal hydrolase L1 in breast carcinoma and its biological significance. Hum Pathol (2013) 44(9):1838–48. 10.1016/j.humpath.2013.02.006 23664488

[45] Jin C Yu W Lou X Zhou F Han X Zhao N UCHL1 is a putative tumor suppressor in ovarian cancer cells and contributes to cisplatin resistance. J Cancer (2013) 4(8):662–70. 10.7150/jca.6641 24155778 PMC3805994

[46] Hussain S Foreman O Perkins SL Witzig TE Miles RR van Deursen J The de-ubiquitinase UCH-L1 is an oncogene that drives the development of lymphoma *in vivo* by deregulating PHLPP1 and Akt signaling. Leukemia (2010) 24(9):1641–55. 10.1038/leu.2010.138 20574456 PMC3236611

[47] Xiang T Li L Yin X Yuan C Tan C Su X The ubiquitin peptidase UCHL1 induces G0/G1 cell cycle arrest and apoptosis through stabilizing p53 and is frequently silenced in breast cancer. PloS one (2012) 7(1):e29783. 10.1371/journal.pone.0029783 22279545 PMC3261155

[48] Finnerty BM Moore M Verma A Aronova A Huang S Edwards DP UCHL1 loss alters the cell-cycle in metastatic pancreatic neuroendocrine tumors. Endocr Relat Cancer (2019) 26(4):411–23. 10.1530/erc-18-0507 30689542

[49] Bergsagel PL Kuehl WM Zhan F Sawyer J Barlogie B Shaughnessy J Jr. Cyclin D dysregulation: an early and unifying pathogenic event in multiple myeloma. Blood (2005) 106(1):296–303. 10.1182/blood-2005-01-0034 15755896 PMC1895118

[50] Mulligan G Mitsiades C Bryant B Zhan F Chng WJ Roels S Gene expression profiling and correlation with outcome in clinical trials of the proteasome inhibitor bortezomib. Blood (2007) 109(8):3177–88. 10.1182/blood-2006-09-044974 17185464

[51] Liu Y Lashuel HA Choi S Xing X Case A Ni J Discovery of inhibitors that elucidate the role of UCH-L1 activity in the H1299 lung cancer cell line. Chem Biol (2003) 10(9):837–46. 10.1016/j.chembiol.2003.08.010 14522054

