# Supplementary material for: Targeting UCHL1 Induces Cell Cycle Arrest in High-Risk Multiple Myeloma with t(4;14)
Source: Pathol Oncol Res. 2021 Mar 31;27:606567. doi: 10.3389/pore.2021.606567 (PMC8262241; doi:10.3389/pore.2021.606567)
Supplement: Supplementary file 1 [file Table1.DOCX]

**Additional information**

| **Supplementary Table** **1.** Details specific to the top 50 upregulated genes | | | | | | |
| --- | --- | --- | --- | --- | --- | --- |
| **No.** | **Gene symbol** | **High-risk**  **average (log2)** | **Standard-risk average (log2)** | **Fold change** | ***p*-value** | **FDR**  ***p*-value** |
| 1 | UCHL1 | 15.02 | 5.80 | 593.15 | 0.02 | 0.23 |
| 2 | GABRB2 | 9.68 | 5.55 | 17.54 | 0.00 | 0.08 |
| 3 | NGFRAP1 | 13.20 | 9.20 | 16.01 | 0.01 | 0.23 |
| 4 | PKP2 | 11.45 | 7.64 | 14.00 | 0.00 | 0.07 |
| 5 | CCND2 | 14.34 | 11.00 | 10.10 | 0.02 | 0.25 |
| 6 | GGH | 11.25 | 8.36 | 7.44 | 0.00 | 0.07 |
| 7 | KCNN3 | 13.43 | 10.54 | 7.38 | 0.01 | 0.23 |
| 8 | WHSC1 | 11.41 | 8.56 | 7.18 | 0.00 | 0.10 |
| 9 | CENPW | 9.48 | 6.67 | 7.01 | 0.00 | 0.01 |
| 10 | RRM2 | 8.27 | 5.51 | 6.79 | 0.00 | 0.17 |
| 11 | POLE2 | 8.50 | 5.76 | 6.68 | 0.00 | 0.04 |
| 12 | MYBL1 | 8.26 | 5.55 | 6.54 | 0.01 | 0.19 |
| 13 | UBE2T | 11.00 | 8.30 | 6.51 | 0.01 | 0.22 |
| 14 | OXCT1 | 11.10 | 8.42 | 6.43 | 0.01 | 0.22 |
| 15 | PLAG1 | 7.91 | 5.29 | 6.14 | 0.00 | 0.15 |
| 16 | ATP2B4 | 12.53 | 9.91 | 6.12 | 0.00 | 0.15 |
| 17 | CSNK1G1 | 9.62 | 7.21 | 5.32 | 0.00 | 0.17 |
| 18 | ADAMTS1 | 7.72 | 5.41 | 4.98 | 0.02 | 0.24 |
| 19 | SNRPD3 | 8.90 | 6.58 | 4.97 | 0.00 | 0.07 |
| 20 | ALDH1L2 | 9.72 | 7.42 | 4.94 | 0.00 | 0.16 |
| 21 | FCRLA | 13.47 | 11.17 | 4.91 | 0.03 | 0.27 |
| 22 | WEE1 | 7.90 | 5.64 | 4.81 | 0.00 | 0.15 |
| 23 | MYC | 9.60 | 7.34 | 4.78 | 0.01 | 0.22 |
| 24 | SLCO4C1 | 7.97 | 5.74 | 4.68 | 0.01 | 0.22 |
| 25 | DPM3 | 11.78 | 9.55 | 4.68 | 0.02 | 0.23 |
| 26 | SLC16A7 | 8.72 | 6.50 | 4.65 | 0.00 | 0.07 |
| 27 | ACSM3 | 9.25 | 7.06 | 4.59 | 0.01 | 0.19 |
| 28 | MKI67 | 9.00 | 6.81 | 4.56 | 0.00 | 0.07 |
| 29 | ITGB1BP1 | 8.69 | 6.51 | 4.55 | 0.00 | 0.16 |
| 30 | HMGN5 | 9.00 | 6.82 | 4.53 | 0.00 | 0.09 |
| 31 | COX4I1 | 11.86 | 9.69 | 4.51 | 0.01 | 0.22 |
| 32 | PNP | 11.41 | 9.24 | 4.48 | 0.00 | 0.17 |
| 33 | ITM2A | 12.56 | 10.42 | 4.38 | 0.01 | 0.20 |
| 34 | PCNA | 11.72 | 9.60 | 4.34 | 0.00 | 0.13 |
| 35 | ETFB | 11.80 | 9.68 | 4.34 | 0.03 | 0.26 |
| 36 | ENPP2 | 8.73 | 6.65 | 4.23 | 0.01 | 0.22 |
| 37 | C15orf41 | 9.33 | 7.25 | 4.21 | 0.00 | 0.14 |
| 38 | INSIG2 | 12.22 | 10.15 | 4.19 | 0.00 | 0.09 |
| 39 | FBXO5 | 7.62 | 5.57 | 4.14 | 0.00 | 0.10 |
| 40 | PRCC | 11.84 | 9.80 | 4.13 | 0.00 | 0.07 |
| 41 | C1orf21 | 10.02 | 7.99 | 4.11 | 0.03 | 0.27 |
| 42 | TAP1 | 12.69 | 10.65 | 4.10 | 0.01 | 0.19 |
| 43 | MRPS14 | 14.74 | 12.71 | 4.09 | 0.00 | 0.12 |
| 44 | GABARAPL2 | 10.07 | 8.04 | 4.08 | 0.01 | 0.23 |
| 45 | FCRL2 | 10.05 | 8.02 | 4.08 | 0.02 | 0.25 |
| 46 | AP1S2 | 10.16 | 8.16 | 4.01 | 0.00 | 0.11 |
| 47 | CDK1 | 8.26 | 6.27 | 3.98 | 0.00 | 0.09 |
| 48 | RMDN1 | 11.31 | 9.32 | 3.98 | 0.00 | 0.07 |
| 49 | HIST1H3B | 11.25 | 9.26 | 3.97 | 0.02 | 0.24 |
| 50 | MIF | 15.38 | 13.40 | 3.94 | 0.02 | 0.25 |

**Abbreviation:** FDR, false discovery rate

| **Supplementary Table** **2.** Details specific to the top 50 downregulated genes | | | | | | |
| --- | --- | --- | --- | --- | --- | --- |
| **No.** | **Gene symbol** | **High-risk**  **average (log2)** | **Standard-risk**  **average (log2)** | **Fold change** | ***p*-value** | **FDR**  ***p*-value** |
| 1 | RGS1 | 7.534 | 10.674 | -8.810 | 0.013 | 0.224 |
| 2 | MLLT3 | 11.876 | 15.002 | -8.734 | 0.003 | 0.158 |
| 3 | QPCT | 10.514 | 13.232 | -6.582 | 0.013 | 0.224 |
| 4 | OR1S2 | 7.112 | 9.790 | -6.396 | 0.009 | 0.208 |
| 5 | NR4A1 | 8.087 | 10.680 | -6.033 | 0.017 | 0.235 |
| 6 | AGR2 | 6.527 | 9.027 | -5.658 | 0.002 | 0.148 |
| 7 | GOLGA8G | 6.688 | 9.183 | -5.638 | 0.008 | 0.201 |
| 8 | ADAMTS16 | 6.285 | 8.727 | -5.434 | 0.001 | 0.122 |
| 9 | PDE4B | 9.545 | 11.957 | -5.325 | 0.001 | 0.089 |
| 10 | ATP10B | 6.724 | 8.980 | -4.777 | 0.014 | 0.228 |
| 11 | GOLGA8G | 8.031 | 10.284 | -4.767 | 0.017 | 0.234 |
| 12 | ERC2 | 5.533 | 7.705 | -4.507 | 0.011 | 0.216 |
| 13 | EGR1 | 7.210 | 9.377 | -4.490 | 0.046 | 0.298 |
| 14 | PRSS3 | 7.110 | 9.244 | -4.388 | 0.018 | 0.235 |
| 15 | CHMP7 | 11.375 | 13.479 | -4.301 | 0.027 | 0.262 |
| 16 | NCOA3 | 11.508 | 13.542 | -4.096 | 0.018 | 0.236 |
| 17 | PIP5K1B | 9.418 | 11.413 | -3.986 | 0.014 | 0.226 |
| 18 | FAM49A | 9.560 | 11.551 | -3.977 | 0.008 | 0.198 |
| 19 | MEF2A | 10.591 | 12.464 | -3.663 | 0.025 | 0.256 |
| 20 | OR4F6 | 5.522 | 7.387 | -3.644 | 0.009 | 0.208 |
| 21 | OR1D4 | 7.447 | 9.311 | -3.641 | 0.016 | 0.231 |
| 22 | OR7E24 | 6.191 | 8.055 | -3.640 | 0.008 | 0.200 |
| 23 | CD79A | 8.469 | 10.328 | -3.629 | 0.042 | 0.289 |
| 24 | OR9G1 | 7.675 | 9.495 | -3.529 | 0.035 | 0.275 |
| 25 | MOXD1 | 6.646 | 8.462 | -3.521 | 0.047 | 0.298 |
| 26 | TLR10 | 6.530 | 8.343 | -3.516 | 0.010 | 0.208 |
| 27 | GOLGA8N | 7.096 | 8.898 | -3.487 | 0.041 | 0.287 |
| 28 | LSP1P3 | 6.940 | 8.733 | -3.466 | 0.019 | 0.238 |
| 29 | UBA7 | 6.962 | 8.750 | -3.454 | 0.010 | 0.208 |
| 30 | GNG2 | 8.142 | 9.925 | -3.443 | 0.003 | 0.162 |
| 31 | GAS8 | 9.845 | 11.618 | -3.417 | 0.042 | 0.290 |
| 32 | LOC442028 | 4.393 | 6.158 | -3.400 | 0.005 | 0.189 |
| 33 | RNASEK | 13.784 | 15.535 | -3.365 | 0.011 | 0.215 |
| 34 | CCDC80 | 6.509 | 8.229 | -3.296 | 0.005 | 0.189 |
| 35 | CDC42EP3 | 5.581 | 7.301 | -3.294 | 0.005 | 0.186 |
| 36 | ARHGEF38 | 6.019 | 7.737 | -3.290 | 0.006 | 0.193 |
| 37 | DOCK10 | 6.092 | 7.801 | -3.270 | 0.010 | 0.210 |
| 38 | OR51L1 | 6.633 | 8.340 | -3.265 | 0.007 | 0.195 |
| 39 | OR4F4 | 6.857 | 8.560 | -3.256 | 0.014 | 0.228 |
| 40 | SNX31 | 5.956 | 7.632 | -3.196 | 0.030 | 0.266 |
| 41 | ZNF841 | 5.611 | 7.284 | -3.188 | 0.021 | 0.246 |
| 42 | OR2M3 | 6.311 | 7.970 | -3.159 | 0.044 | 0.294 |
| 43 | TMEM123 | 13.193 | 14.843 | -3.139 | 0.001 | 0.119 |
| 44 | OR52N5 | 4.658 | 6.309 | -3.139 | 0.008 | 0.201 |
| 45 | LGALS16 | 6.656 | 8.282 | -3.088 | 0.005 | 0.188 |
| 46 | CUL4A | 7.327 | 8.948 | -3.076 | 0.003 | 0.154 |
| 47 | TMEM217 | 6.037 | 7.653 | -3.065 | 0.016 | 0.232 |
| 48 | SAMD4B | 11.111 | 12.713 | -3.035 | 0.004 | 0.176 |
| 49 | SET | 9.800 | 11.397 | -3.025 | 0.049 | 0.303 |
| 50 | SLC38A9 | 6.758 | 8.355 | -3.025 | 0.013 | 0.224 |

**Abbreviation:** FDR, false discovery rate

| **Supplementary Table 3.** Enriched gene sets from Gene Set Enrichment Analysis (GSEA) | | | | |
| --- | --- | --- | --- | --- |
| **NO.** | **GS** | **SIZE** | **NOM**  ***p*-value** | **FDR**  **q-value** |
|  | **follow link to MSigDB** |  |  |  |
| 1 | HALLMARK_MTORC1_SIGNALING | 194 | < 0.001 | 0.081 |
| 2 | HALLMARK_MITOTIC_SPINDLE | 194 | < 0.001 | 0.080 |
| 3 | HALLMARK_G2M_CHECKPOINT | 188 | < 0.001 | 0.093 |
| 4 | HALLMARK_MYC_TARGETS_V1 | 178 | 0.002 | 0.073 |
| 5 | HALLMARK_OXIDATIVE_PHOSPHORYLATION | 191 | 0.003 | 0.093 |
| 6 | HALLMARK_FATTY_ACID_METABOLISM | 154 | 0.004 | 0.071 |
| 7 | HALLMARK_E2F_TARGETS | 185 | 0.007 | 0.084 |
| 8 | HALLMARK_GLYCOLYSIS | 192 | 0.017 | 0.076 |
| 9 | HALLMARK_PROTEIN_SECRETION | 93 | 0.02 | 0.096 |
| 10 | HALLMARK_DNA_REPAIR | 135 | 0.023 | 0.088 |
| 11 | HALLMARK_ADIPOGENESIS | 189 | 0.024 | 0.092 |
| 12 | HALLMARK_UNFOLDED_PROTEIN_RESPONSE | 100 | 0.026 | 0.113 |
| 13 | HALLMARK_PI3K_AKT_MTOR_SIGNALING | 101 | 0.032 | 0.101 |
| 14 | HALLMARK_PEROXISOME | 98 | 0.042 | 0.093 |
| 15 | HALLMARK_HEME_METABOLISM | 188 | 0.046 | 0.123 |
| 16 | HALLMARK_MYC_TARGETS_V2 | 51 | 0.062 | 0.138 |

**Abbreviations:** GS, gene set; NOM, nominal; FDR, false discovery rate

| **Supplementary Table 4.** Details of all enriched gene sets from FUNRICH software | | | | | | |
| --- | --- | --- | --- | --- | --- | --- |
| **Biological pathway** | **Genes in the dataset** | **Genes in the background dataset** | **Percentage of genes** | **Fold enrichment** | ***p*-value** | **Genes mapped**  **(from input data set)** |
| Ubiquitin-dependent degradation of Cyclin D | 11 | 66 | 2.94 | 2.81 | 0.00158 | PSMB2; CDK4; PSMB9; PSMB8; PSMD11; PSMD10; PSMD4; UBE2C; PSMA2; PSMA3; and, PSMD14 |
| Signaling by Wnt | 12 | 75 | 3.21 | 2.69 | 0.00141 | PSMB2; PSMB9; PSMB8; PSMD11; PSMD10; PSMD4; UBE2C; GSK3B; PSMA2; PSMA3; PSMD14; and, AXIN1 |
| E2F-mediated regulation of DNA replication | 7 | 28 | 1.87 | 4.21 | 0.00099 | RRM2; PCNA; FBXO5; CDK1; CCNB1; MCM8; and, E2F1 |
| Cyclin A:Cdk2-associated events at S phase entry | 15 | 90 | 4.01 | 2.80 | 0.00023 | WEE1; CDK1; CKS1B; PSMB2; PSMB9; MCM8; PSMB8; PSMD11; MCM6; PSMD10; PSMD4; UBE2C; PSMA2; PSMA3; and, PSMD14 |
| Cdc20:Phospho-APC/C-mediated degradation of Cyclin A | 13 | 69 | 3.48 | 3.17 | 0.00017 | CDK1; MAD2L1; PSMB2; PSMB9; PSMB8; PSMD11; PSMD10; PSMD4; UBE2C; PSMA2; PSMA3; BUB3; and, PSMD14 |
| The citric acid (TCA) cycle and respiratory electron transport | 19 | 118 | 5.08 | 2.71 | 0.00006 | COX4I1; ETFB; NDUFB10; ATP5G1; NDUFA6; COX5B; SUCLG1; UQCRC2; NDUFC1; PDHB; SDHB; UQCR10; SDHC; ATP5J; NDUFA5; COX6C; MDH2; NDUFB3; and, NDUFA8 |
| Regulation of APC/C activators between G1/S and early anaphase | 15 | 78 | 4.01 | 3.24 | 0.00004 | FBXO5; CDK1; MAD2L1; PSMB2; CCNB1; PSMB9; PSMB8; PSMD11; PSMD10; PSMD4; UBE2C; PSMA2; PSMA3; BUB3; and, PSMD14 |
| DNA replication | 33 | 261 | 8.82 | 2.13 | 0.00003 | POLE2; PCNA; FBXO5; CDK1; ITGB3BP; MAD2L1; DCTN3; PSMB2; PSMB9; MCM8; CENPP; PSMB8; PSMD11; CENPF; MCM6; PSMD10; GMNN; PSMD4; SGOL1; UBE2C; E2F1; SPDL1; CENPN; PSMA2; PSMA3; CETN2; FGFR1OP; BUB3; XPO1; BUB1; CDC7; PSMD14; and, KIF20A |
| Cell cycle checkpoints | 20 | 118 | 5.35 | 2.85 | 0.00002 | WEE1; CDK1; MAD2L1; PSMB2; CCNB1; PSMB9; MCM8; PSMB8; CCNB2; PSMD11; MCM6; PSMD10; PSMD4; UBE2C; PSMA2; PSMA3; BUB3; CDC7; PSMD14; and, RAD9B |
| G2/M DNA replication checkpoint | 4 | 4 | 1.07 | 16.82 | 0.00001 | WEE1; CDK1; CCNB1; and, CCNB2 |

**Supplementary Table 5.** Functional enrichments linked to protein-protein interaction (PPI) network

| **GO-term** | **Description** | **FDR** |
| --- | --- | --- |
| GO:0032268 | Regulation of cellular protein metabolic process | 2.23e-06 |
| GO:0060070 | Canonical Wnt signaling pathway | 7.77e-05 |
| GO:0045859 | Regulation of protein kinase activity | 7.77e-05 |
| GO:0090068 | Positive regulation of cell cycle process | 9.60e-05 |
| GO:0045737 | Positive regulation of cyclin-dependent protein serine/threonine kinase activity | 0.00017 |
| GO:2000045 | Regulation of G1/S transition of mitotic cell cycle | 0.00019 |
| GO:0051726 | Regulation of cell cycle | 0.00023 |

**Abbreviations:** GO, Gene ontology; FDR, false discovery rate


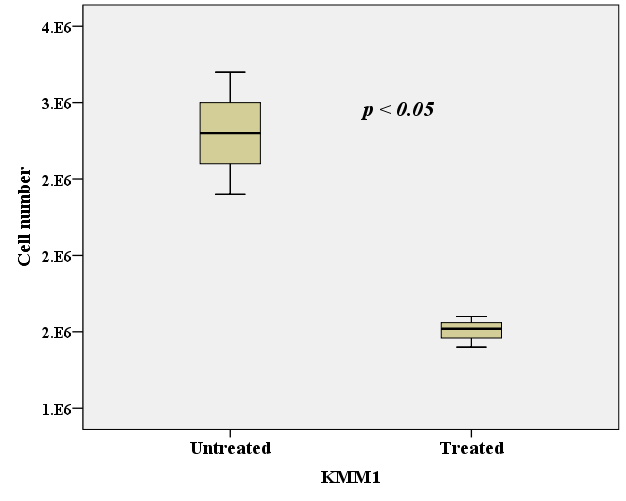

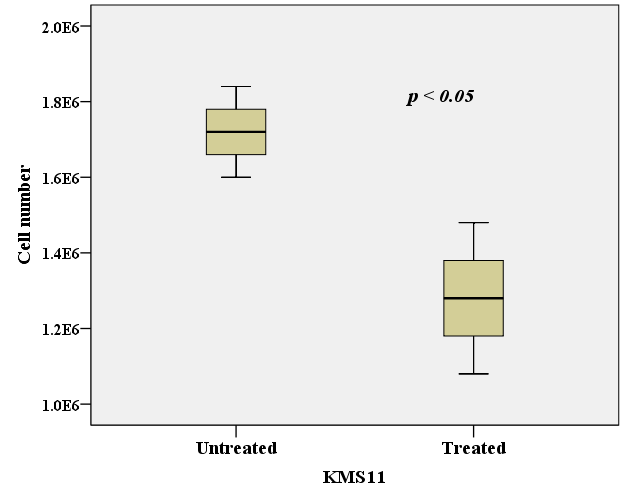


**S1A**

**S1B**

**Figure S1** Cell counting were performed using trypan blue staining. The results have revealed that cell numbers of (B) KMM1 and (B) KMS11 have been decreased after treated with UCHL1 inhibitor. These results explored the antiproliferative effect from UCHL1 inhibitor.


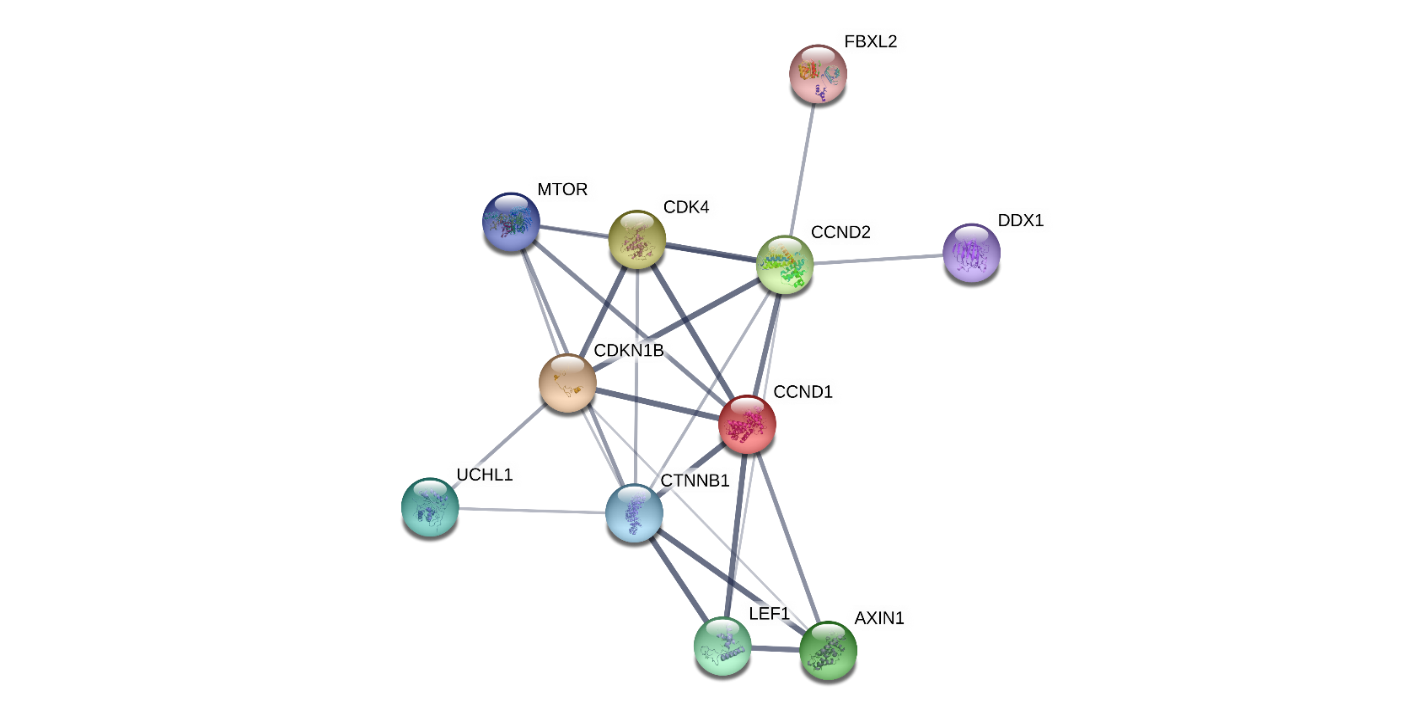

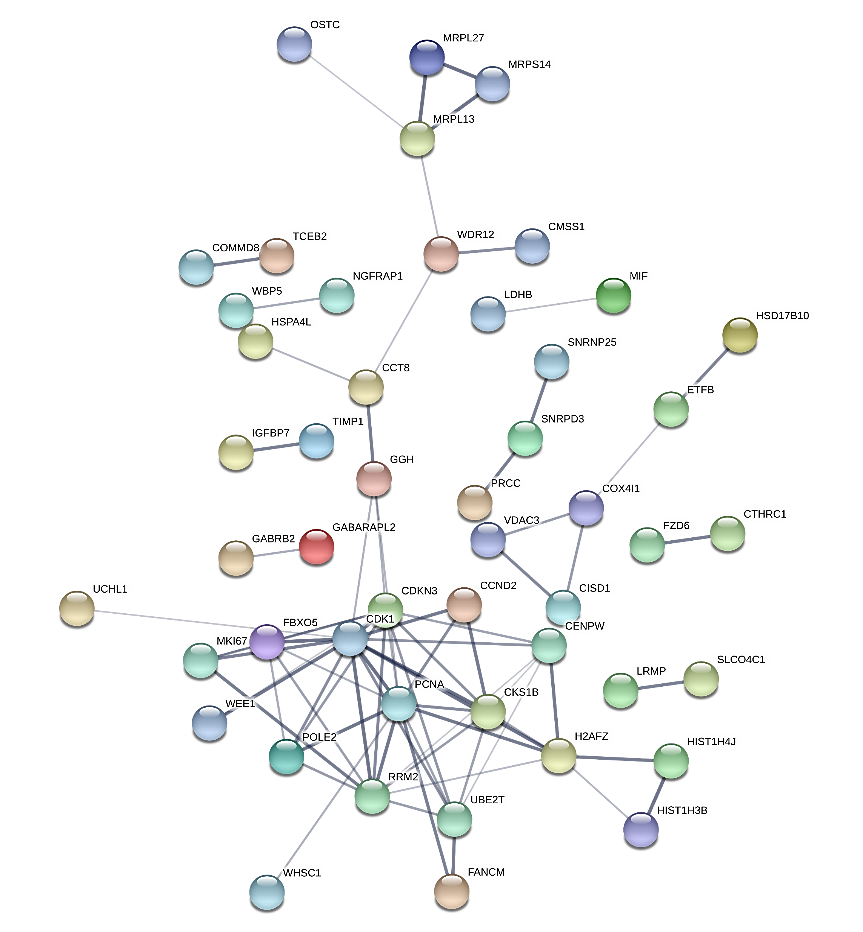


**S2A**

**S2B**

**Figure S2** (A) The protein-protein interaction (PPI) network of UCHL1 and its well-known downstream targets from previous studies indicates that UCHL1 can function via downstream targets, including CCND2, which is the downstream target we found in this study. (B) PPI network of the top 100 upregulated genes from expression profile analysis in this study demonstrated that UCHL1 is able to function via CCND2 molecular network involvement as in red circle. CCND2 was the only downstream target identified in the PPI network in this study.
